# INFINITy: A fast machine learning‐based application for human influenza A and B virus subtyping

**DOI:** 10.1111/irv.13096

**Published:** 2023-01-25

**Authors:** Marco Cacciabue, Débora N. Marcone

**Affiliations:** ^1^ Instituto de Agrobiotecnología y Biología Molecular (IABIMO), Instituto Nacional de Tecnología Agropecuaria (INTA) Hurlingham Argentina; ^2^ Consejo Nacional de Investigaciones Científicas y Técnicas (CONICET) Buenos Aires Argentina; ^3^ Departamento de Ciencias Básicas Universidad Nacional de Luján Luján Argentina; ^4^ Cátedra de Virología, Instituto de Bacteriología y Virología Molecular (IBaViM), Facultad de Farmacia y Bioquímica Universidad de Buenos Aires Buenos Aires Argentina; ^5^ Cátedra de Microbiología, Parasitología y Virología, Facultad de Ciencias Médicas Pontificia Universidad Católica Argentina Buenos Aires Argentina

**Keywords:** clades, genetic groups, hemagglutinin, influenza, machine learning, sequence, subclades, subtyping

Influenza viruses are one of the main agents causing acute respiratory infections (ARI) in humans resulting in a large amount of illness and death globally.^1^, ^2^ The influenza viruses classification is based on the nomenclature proposed by the World Health Organization (WHO)^3^ that is widely accepted and used by the medical and scientific communities throughout the world. Since the pandemic in 2009, two subtypes of human influenza A viruses, A(H1N1)pdm09 and A(H3N2), and two lineages of influenza B, B/Victoria and B/Yamagata, have been responsible for the vast majority of cases each year. Within each subtype and lineage, different clades and genetic groups were described to reflect the continuous viral evolution, driven by antigenic drift. The WHO Global Influenza Surveillance and Response System (GISRS) studies human influenza viruses from >110 countries, to monitor circulating strains, understand epidemiology and evolution, and contribute to verify the vaccine effectiveness and update its formulation each year.^4^, ^5^ A growing number of laboratories and research centers is contributing to this initiative by sequencing the whole viral genome or the hemagglutinin (HA) gene from local strains.

Influenza clade classification is usually performed by phylogenetic analysis of HA gene sequences from circulating strains along with reference sequences, which is a time‐consuming process and requires specific training and equipment. Alternatively, this can be done by comparing amino acid substitutions, either manually or by using in‐house scripts. While there are currently specific tools available for influenza classification,^6^, ^7^, ^8^ they have several limitations such as: (a) they require an alignment of the input data against reference sequences (which can be computationally expensive), (b) requirement of multiple ad hoc programs installed, (c) users should be familiar with the command line, (d) users must create a template containing clade‐defining amino acid pattern by position, (e) only classifies sequences into type A or B and subtype/lineage but cannot discern clades or genetic groups, and (f) take into account only the most prevalent and recent influenza clades.

Advanced machine learning techniques have proven to make accurate predictions, using algorithms that reveal patterns in large datasets. In the analysis of viral data, machine learning methods have been recently implemented, for example, in: COVIDEX, a tool that classifies complete genome nucleotide sequences of SARS‐CoV‐2 into lineages,^9^ a recent application for avian influenza clade classification,^10^ the prediction of phenotypes for human influenza A from proteomic input,^11^ and detection of new variants using ensemble learning.^12^

In this sense, we developed INFINITy, a tool based on alignment‐free machine learning for human influenza virus classification into subtypes and clades. INFINITy is a web application that runs on an internet connection without any installation and has a user‐friendly interface. It is fast, sensitive, specific, and ready to implement. Additionally, it is available to run locally for R and Rstudio users as an R package. Furthermore, two docker images are available to secure the reproducibility of the results.

INFINITy includes two classification models: one for complete HA sequences (FULL HA, for whole gene sequence length of 1700 bp) and other for the HA1 subunit coding sequence (HA1, for the initial 1030 bp of the HA gene). The influenza classification comprises 75 clades or genetic groups: 25 for A(H1N1)pdm09, 32 for A(H3N2), and 14 for B/Victoria and 4 for B/Yamagata (supporting information Table S1).

The overall classification algorithm is divided into three phases:

1. The first phase loads the user data in a multifasta format and performs the k‐mer counting operation using the k‐mer package.^13^ Each k‐mer count is normalized over the k‐mer size (*k* = 6) and the sequence length.
2. The second phase calls the ranger package^14^ predict function using one of the two pre‐trained random forest models (FULL HA or HA1) and obtains a probability score based on the rule of majority vote. From this, the app obtains the score for each query sequence classification, the proportion of N bases in the genome, and the genome length.
3. Finally, two tables are created, one showing the sequences that passed all the quality checks and another with sequences that did not pass some of the filter steps. These filters controls: that each sequence obtained a probability score of 0.4 or more, that the sequence length is close to the expected sequence length for the classification model (FULL HA 1700 or HA1 1030) for a factor of no more that 50%, and that the percentage of ambiguous bases in the sequence (N) is not larger than 2%. A brief report can be produced including the results table, date of analysis, and model information (Figure 1).

**FIGURE 1:**
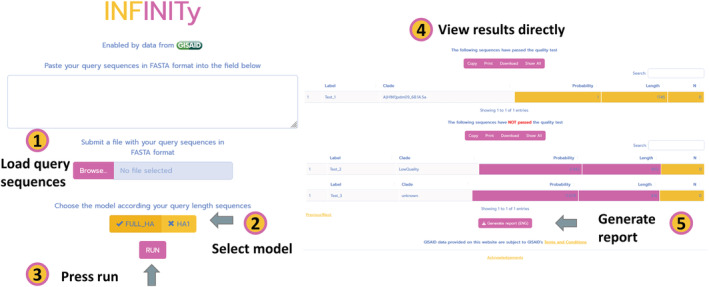
Overview of the INFINITy application. The user loads a sequence file, or copy and paste the sequences, selects the corresponding model, and presses RUN. Two results tables will be shown, showing the sequences that passed the quality controls and those that did not. Although all sequences are classified, the user should carefully interpret the results considering the quality control for each one. Sequences that did NOT passed the quality filters are shown as “LowQuality”, and those sequences with a probability score below a value of 0.2 are shown as “unknown”. Finally, the user can download an automatic report.

In order to train the classification models, a reference dataset was created by downloading complete HA sequences of influenza A(H1N1)pdm09, A(H3N2), B/Victoria, and B/Yamagata from GISAID. We defined the influenza clade and subclade for each sequence by analyzing their amino acid composition and the combination of signature position mutations, according to the WHO nomenclature. A phylogenetic analysis by influenza type and lineage was used to confirm the classification. The final HA gene dataset includes a total of 11,316 sequences with an average length of 1700 bp: 2957 influenza A(H1N1)pdm09, 3112 A(H3N2), 2963 B/Victoria, and 2284 B/Yamagata sequences. To generate a dataset of the HA1 region, complete HA sequences were cut at nucleotide position 1030. For each dataset, FULL HA or HA1, we developed a specific classification model. For each model, a subset (training dataset) of approximately 80% of the sequences from each subclade was randomly selected and used to train the random forest model (1000 trees). The remaining 20% of the sequences constituted the testing dataset which was used to evaluate the performance of the respective model.

Both models performed very well, with an accuracy of 0.9952 and 0.9931 for FULL HA and HA1 models, respectively. Additionally, the multiclass AUC was 0.9994 in both cases (supporting information Table S2). Correlation heatmaps, metrics tables, precision‐recall curves, and other statistics were generated for each model (supporting information File S1).

To use the app, the user only loads the input file, a FASTA file with unaligned influenza HA or HA1 gene segment query sequences, selects one of the models according to the length of the query sequences (FULL HA or HA1), and presses the run button (Figure 1). To obtain the most accurate results, we recommend using sequences with a proportion of N bases <1%. Since the HA gene allows for more accurate predictions for subtyping based on phylogeny or machine learning models, the other seven influenza genomic segments were not considered in this version but could be incorporated in the future.

Due to the increasing number of laboratories and researchers using sequencing technologies applied to molecular epidemiology, there is an increasing need of easier and faster applications that allows an accurate and specific classification of viral sequences with no need for specialized training. This is particularly relevant for respiratory pathogens such as influenza viruses that cause annual epidemics with up to 60 million ARI cases worldwide and require a continuous monitoring of circulating strains, which is why we believe INFINITy can help researchers working on this area.

## AUTHOR CONTRIBUTIONS

**Marco Cacciabue:** Formal analysis; methodology; software; validation; visualization; writing‐review and editing. **Débora N. Marcone**: conceptualization, investigation, data curation, supervision, validation, visualization, writing – original draft preparation, review & editing.

## CONFLICT OF INTEREST

Authors declare no conflict of interest.

## PERMISSION TO REPRODUCE MATERIAL FROM OTHER SOURCES

All data are available at GISAID Influenza database.

## Supporting information


**Table S1.** Influenza A & B clades composition

**Table S2.** Classification models stats.

**File S1.** This file contains performance statistics of the two classification models: FULL HA and HA1.

## Data Availability

The application code and instructions are available via Github (https://github.com/marcocacciabue/infinity). Additionally, the web application is available without installation (https://infinity.unlu.edu.ar/).
