# Supplementary material for: INFINITy: A fast machine learning‐based application for human influenza A and B virus subtyping
Source: Influenza Other Respir Viruses. 2023 Jan 25;17(1):e13096. doi: 10.1111/irv.13096 (PMC9874948; doi:10.1111/irv.13096)
Supplement: Supplementary file 1 — Table S1. Influenza A & B clades composition [file IRV-17-e13096-s001.docx]

**Supplementary Table 1. Influenza A & B clades composition**

|  | Clades (n) |
| --- | --- |
| **FluA(H1N1)pdm09** | 25 |
| A(H1N1)pdm09_1 |  |
| A(H1N1)pdm09_2 |  |
| A(H1N1)pdm09_3 |  |
| A(H1N1)pdm09_4 |  |
| A(H1N1)pdm09_5 |  |
| A(H1N1)pdm09_6 |  |
| A(H1N1)pdm09_6A |  |
| A(H1N1)pdm09_6B |  |
| A(H1N1)pdm09_6B.1 |  |
| A(H1N1)pdm09_6B.1A |  |
| A(H1N1)pdm09_6B.1A.1 |  |
| A(H1N1)pdm09_6B.1A.2 |  |
| A(H1N1)pdm09_6B.1A.3 |  |
| A(H1N1)pdm09_6B.1A.4 |  |
| A(H1N1)pdm09_6B.1A.5 |  |
| A(H1N1)pdm09_6B.1A.5a |  |
| A(H1N1)pdm09_6B.1A.5a.1 |  |
| A(H1N1)pdm09_6B.1A.5a.2 |  |
| A(H1N1)pdm09_6B.1A.5b |  |
| A(H1N1)pdm09_6B.1A.6 |  |
| A(H1N1)pdm09_6B.1A.7 |  |
| A(H1N1)pdm09_6B.2 |  |
| A(H1N1)pdm09_6C |  |
| A(H1N1)pdm09_7 |  |
| A(H1N1)pdm09_8 |  |
| **FluA(H3N2)** | 32 |
| A(H3N2)_1 |  |
| A(H3N2)_2 |  |
| A(H3N2)_3A |  |
| A(H3N2)_3B |  |
| A(H3N2)_3C |  |
| A(H3N2)_3C.1 |  |
| A(H3N2)_3C.2 |  |
| A(H3N2)_3C.2a |  |
| A(H3N2)_3C.2a1 |  |
| A(H3N2)_3C.2a1a |  |
| A(H3N2)_3C.2a1b |  |
| A(H3N2)_3C.2a1b.1 |  |
| A(H3N2)_3C.2a1b.1a |  |
| A(H3N2)_3C.2a1b.1b |  |
| A(H3N2)_3C.2a1b.2 |  |
| A(H3N2)_3C.2a1b.2a |  |
| A(H3N2)_3C.2a1b.2a.1 |  |
| A(H3N2)_3C.2a1b.2a.2 |  |
| A(H3N2)_3C.2a1b.2b |  |
| A(H3N2)_3C.2a1b.3 |  |
| A(H3N2)_3C.2a2 |  |
| A(H3N2)_3C.2a2/re |  |
| A(H3N2)_3C.2a3 |  |
| A(H3N2)_3C.2a4 |  |
| A(H3N2)_3C.3 |  |
| A(H3N2)_3C.3a |  |
| A(H3N2)_3C.3a.1 |  |
| A(H3N2)_3C.3b |  |
| A(H3N2)_4 |  |
| A(H3N2)_5 |  |
| A(H3N2)_6 |  |
| A(H3N2)_7 |  |
| **FluB/Victoria** | 14 |
| B/Victoria_2 |  |
| B/Victoria_3 |  |
| B/Victoria_4 |  |
| B/Victoria_5 |  |
| B/Victoria_6 |  |
| B/Victoria_V1A |  |
| B/Victoria_V1A.1 |  |
| B/Victoria_V1A.2 |  |
| B/Victoria_V1A.3 |  |
| B/Victoria_V1A.3a |  |
| B/Victoria_V1A.3a.1 |  |
| B/Victoria_V1A.3a.2 |  |
| B/Victoria_V1A+K165N |  |
| B/Victoria_V1B |  |
| **FluB/Yamagata** | 4 |
| B/Yamagata_1 |  |
| B/Yamagata_2 |  |
| B/Yamagata_3 |  |
| B/Yamagata_3+L172Q |  |
