# Supplementary material for: INFINITy: A fast machine learning‐based application for human influenza A and B virus subtyping
Source: Influenza Other Respir Viruses. 2023 Jan 25;17(1):e13096. doi: 10.1111/irv.13096 (PMC9874948; doi:10.1111/irv.13096)
Supplement: Supplementary file 2 — Table S2. Classification models stats. [file IRV-17-e13096-s003.docx]

**Supplementary Table 2**. **Classification models stats.** Basic statistics for each classification model. Accuracy is the proportions of sequences correctly labeled / number of sequences. Multi-class AUC is the mean AUC from all pairwise class comparisons.

| **Model** | **Number of clades** | **Number of trees** | **Training dataset size** | **Testing dataset**  **size** | **Passing** | **Failing** | **Accuracy** | **Multi-class AUC** |
| --- | --- | --- | --- | --- | --- | --- | --- | --- |
| **Full HA** | 75 | 1000 | 9869 | 1447 | 1445 | 2 | 0.9952 | 0.9994 |
| **HA1** | 75 | 1000 | 9869 | 1447 | 1446 | 1 | 0.9931 | 0.9994 |
