# Supplementary material for: INFINITy: A fast machine learning‐based application for human influenza A and B virus subtyping
Source: Influenza Other Respir Viruses. 2023 Jan 25;17(1):e13096. doi: 10.1111/irv.13096 (PMC9874948; doi:10.1111/irv.13096)
Supplement: Supplementary file 3 — File S1. This file contains performance statistics of the two classification models: FULL HA and HA1. [file IRV-17-e13096-s002.docx]

**Supplementary File 1.** This file contains performance statistics of the two classification models: FULL HA and HA1.

INFINITy. FULL HA model

First, we present some basic stats from the training and testing datasets for the FULL HA model.

**Supp. Table 1. Composition of training and testing datasets for the FULL HA model.**

| Classification model | Training date | Training Sequences | Number of subtypes | Number of trees | Mtry* | Oob Error rate** |
| --- | --- | --- | --- | --- | --- | --- |
| Flu | 2022-05-12 | 9869 | 75 | 1000 | 200 | 0.005 |

| Classification model | Testing Sequences | Number of subtypes | Error | Multi-class AUC*** |
| --- | --- | --- | --- | --- |
| Flu | 1447 | 75 | 0.0048 | 0.9994 |

*Mtry is the number of variables randomly sampled as candidates at each split.

****** out-of-bag error is an estimate of the error rate.

*** Multi-class AUC is the mean AUC from all pairwise class comparisons.

Of note: For clade B/Victoria_4, we found only 3 sequences available in public databases. For this reason, all 3 sequences were used for both training and testing.

**Supp. Figure 1. Probability vs the number of ambiguous bases for each sequence tested by INFINITy, using the FULL HA model.**


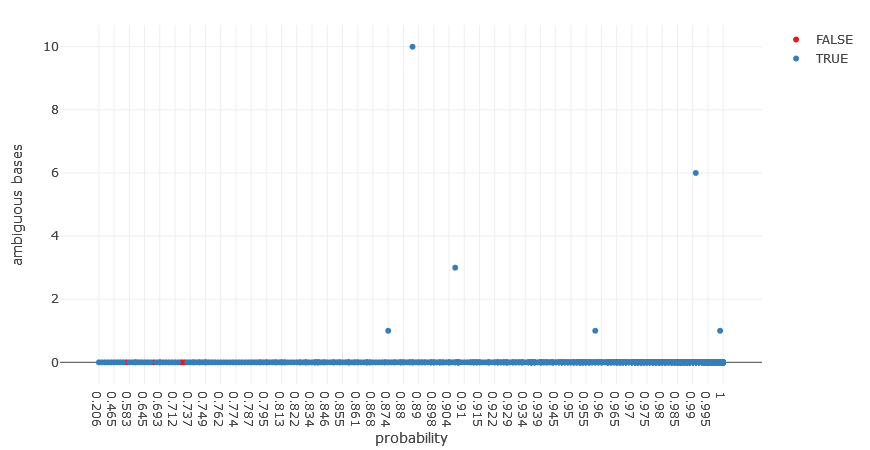
As expected, the proportion of wrongly classified sequences (red dots) increases with lower probability values. Also, we see a trend towards larger proportion of wrongly classified sequences with the number of ambiguous bases.

**Supp. Figure 2. Correlation between the expected classification and the obtained classification obtained by INFINITy for each influenza clade and subclade, using the FULL HA model.**


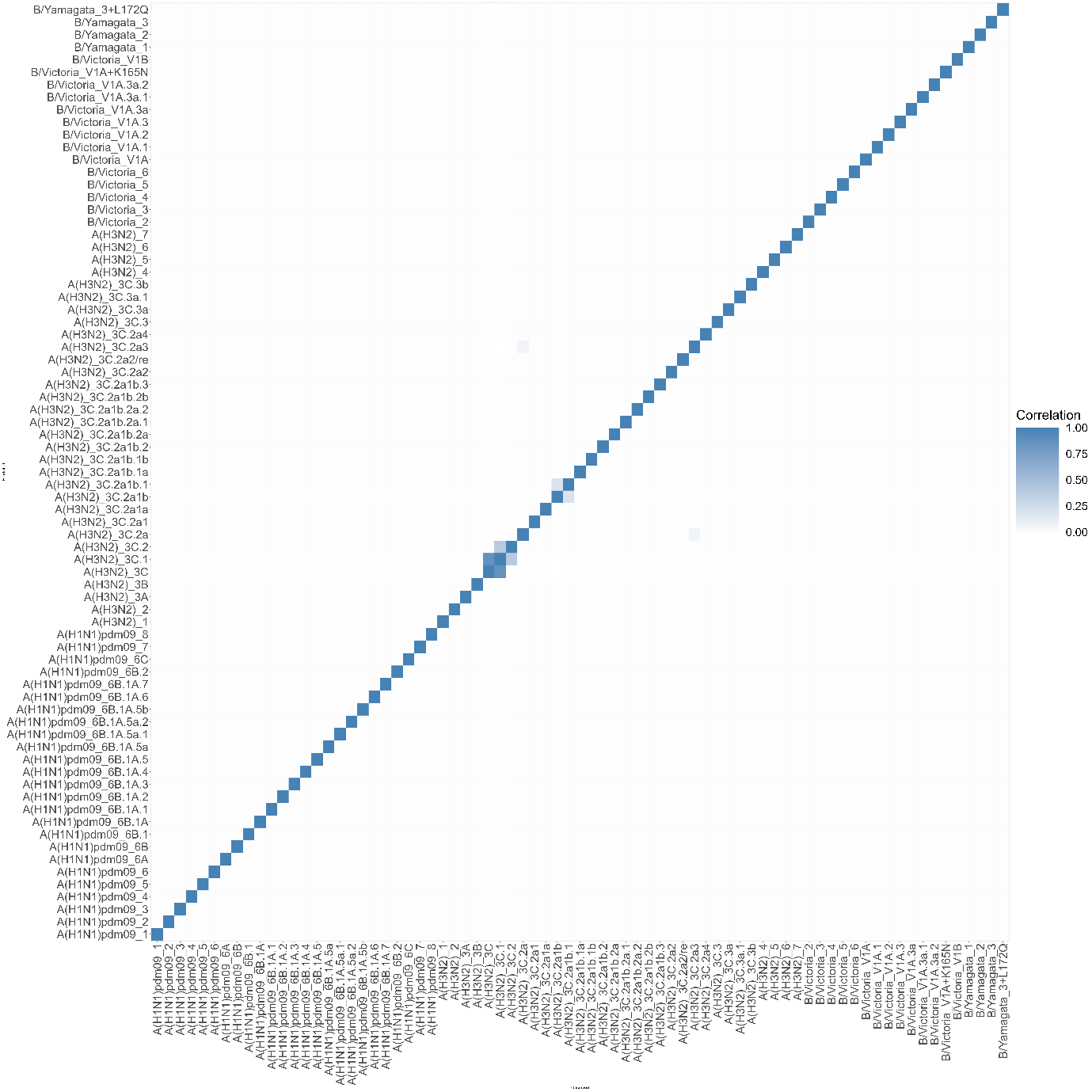


Overall, we find a high correlation value between each influenza type, lineage, clade and subclade.

**Supp. Figure 3. Precision-Recall curve of the FULL HA model.**


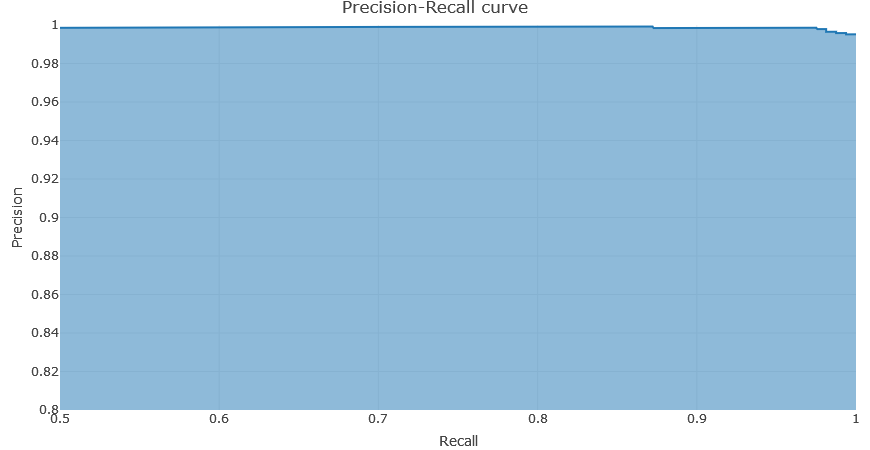
 The Precision-Recall curve shows the good performance of the method.

**Supp. Figure 4. Frequency of probabilities obtained for testing sequences in the INFINITy app, with the FULL HA model**


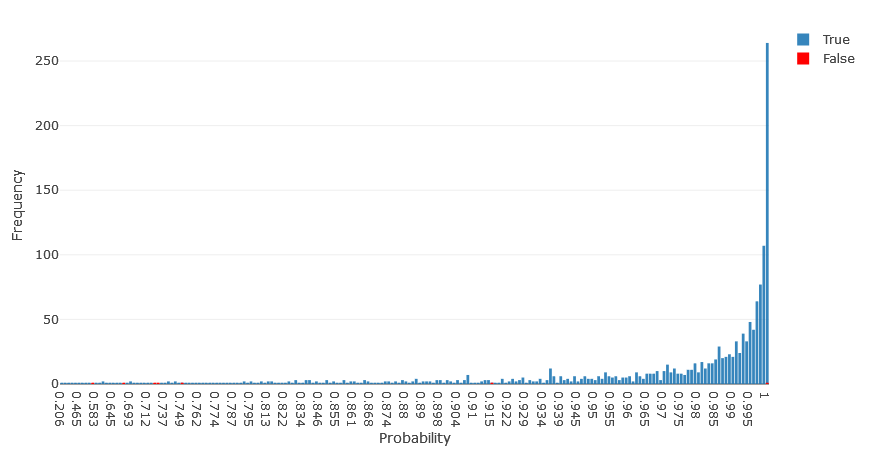


The histogram shows the distribution of the classification across different probability scores, where the highest frequencies are observed for the highest probabilities.

INFINITy. HA1 model

Here we present some basic stats from the training and testing datasets for the HA1 model.

**Supp. Table 2. Composition of training and testing datasets for the HA1 model.**

| Classification model | Training date | Training Sequences | Number of subtypes | Number of trees | Mtry* | Oob Error rate** |
| --- | --- | --- | --- | --- | --- | --- |
| HA1 | 2022-05-20 | 9869 | 75 | 1000 | 200 | 0.0052 |

| Classification model | Testing Sequences | Number of subtypes | Error | Multi-class AUC*** |
| --- | --- | --- | --- | --- |
| HA1 | 1447 | 75 | 0.0069 | 0.9994 |

*Mtry is the number of variables randomly sampled as candidates at each split.

****** out-of-bag error is an estimate of the error rate.

*** Multi-class AUC is the mean AUC from all pairwise class comparisons.

Of note: For clade B/Victoria_4, we found only 3 sequences available in public databases. For this reason, all 3 sequences were used for both training and testing.

**Supp. Figure 5. Probability vs the number of ambiguous bases for each sequence tested by INFINITy, using the HA1 model.**


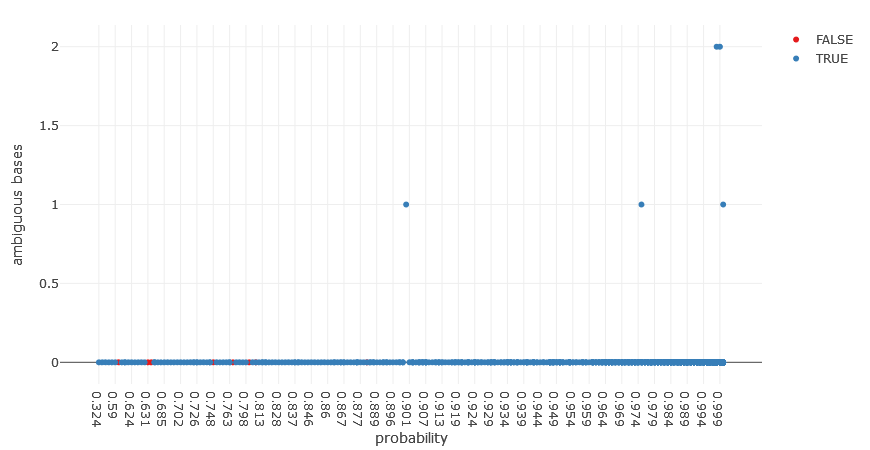
As expected, the proportion of wrongly classified sequences (red dots) increases with lower probability values. Also, we see a trend towards larger proportion of wrongly classified sequences with the number of ambiguous bases.

**Supp. Figure 6. Correlation between the expected classification and the obtained classification obtained by INFINITy for each influenza clade and subclade, using the HA1 model.**


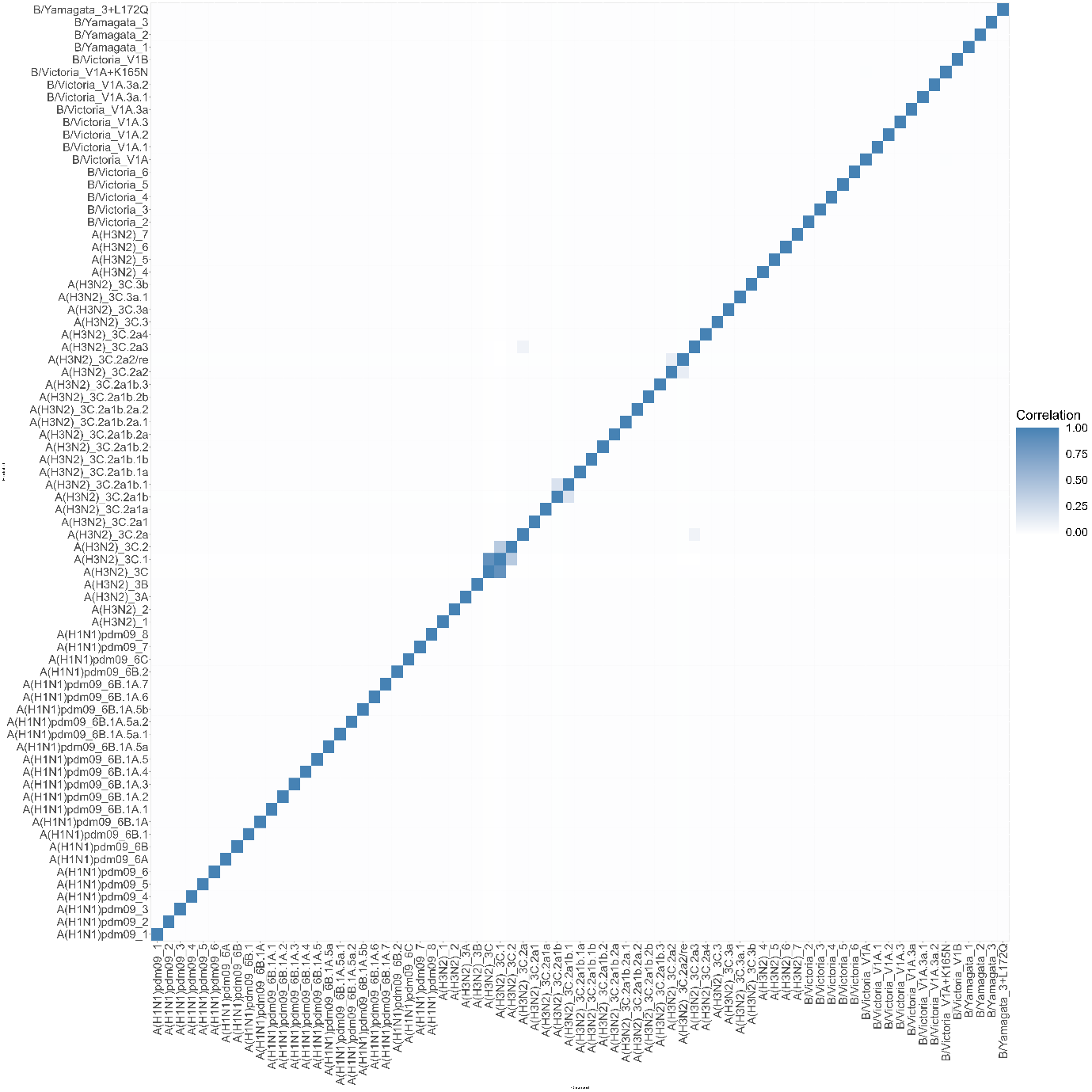


Overall, we find a high correlation value between each influenza type, lineage, clade and subclade.

**Supp. Figure 7. Precision-Recall curve of the HA1 model.**


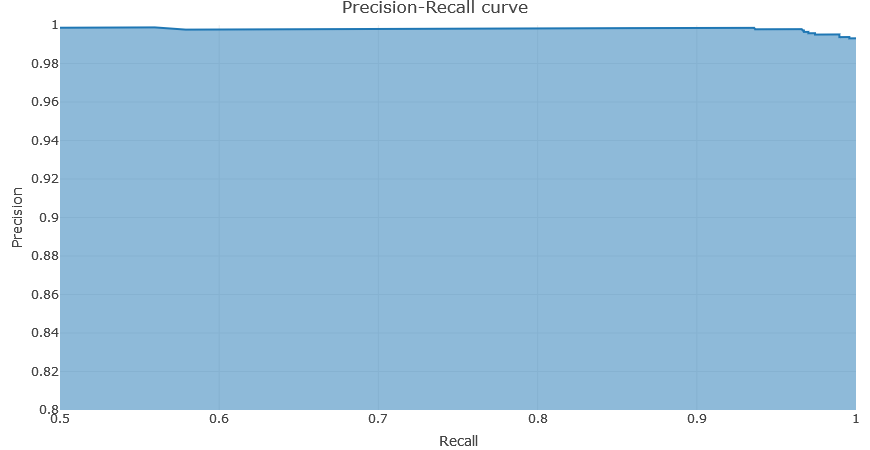
 The Precision-Recall curve shows the good performance of the method.

**Supp. Figure 8. Frequency of probabilities obtained for testing sequences in the INFINITy app, with the HA1 model.**


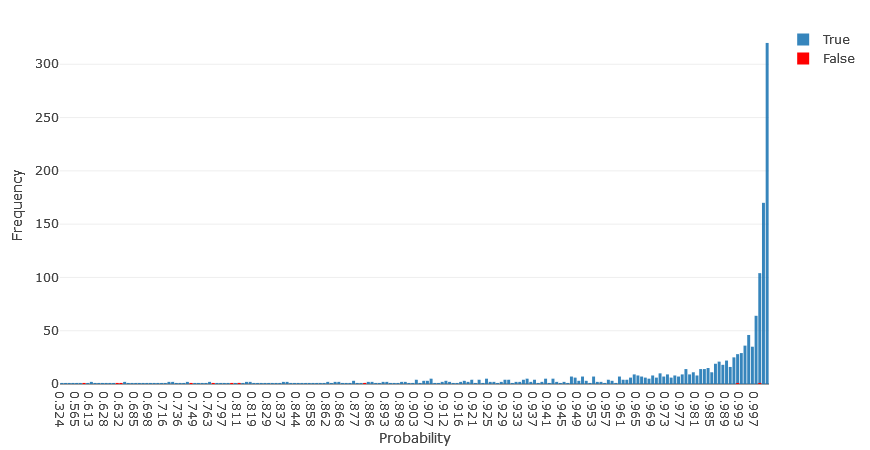
 Finally, the histogram shows the distribution of the classification across different probability scores, where the highest frequencies are observed for the highest probabilities.
